# Additions to Lyophyllaceae s.l. from China

**DOI:** 10.3390/jof7121101

**Published:** 2021-12-20

**Authors:** Jize Xu, Xiaodong Yu, Nakarin Suwannarach, Yi Jiang, Wei Zhao, Yu Li

**Affiliations:** 1 Department of Applied Biological Science, Faculty of Agriculture, Jilin Agriculture Science and Technology University, Jilin 132101, China; jy2920247902@foxmail.com (Y.J.); zhaowei0124@foxmail.com (W.Z.); 2 Department of Protection and Utilization of Wild Animals and Plants, Faculty of Chinese Herbal Medicine, Jilin Agricultural University, Changchun 130118, China; yxd9426@foxmail.com; 3 Research Center of Microbial Diversity and Sustainable Utilization, Faculty of Science, Chiang Mai University, Chiang Mai 50200, Thailand; suwan.462@gmail.com; 4 Faculty of Plant Protection, Jilin Agricultural University, Changchun 130118, China; yuli966@163.com

**Keywords:** *Calocybe*, lyophylloid species, morphological characteristics, phylogenetic analyses, taxonomy

## Abstract

Four new species, viz. *Calocybe coacta*, *C. fulvipes*, *C. vinacea* and *Clitolyophyllum umbilicatum*, are described in northern China. Comparisons are made of macro- and micromorphological features among the new species and closely related species within the genus. The new species feature unique morphological characteristics that separate them from the previously described species. *Calocybe coacta* is characterized by medium- to large-sized basidiocarps, greyish cream, felty pileus and non-cellular epicutis. The key characteristics of *C. fulvipes* are rose-brown to greyish-brown pileus, stone-brown stipe and non-cellular epicutis. The unique morphological characteristics of *C*. *vinacea* that distinguish it from its closely related species are pastel red to dull-red pileus and stipe surface with densely white pruina. The main characteristics of *Clitolyophyllum umbilicatum* are deeply depressed dark orange to light-brown pileus, central stipe and subglobose-ellipsoid spores. Phylogenetic analyses based on the ITS and 28S regions indicated that the four new species are distinct and monophyletic. Full descriptions, color images, illustrations and a phylogenetic tree that show the placement of the four new species are provided. A key to the *Calocybe* species reported from China is also given.

## 1. Introduction

Lyophyllaceae Jülich was suggested by recent molecular phylogenetic analyses to be a putative wider concept that includes several lineages that seem more distantly related, and the generic concepts should be reconsidered [1,2,3,4,5]. Several new genera established in the past ten years were expected to reorganize the system, viz. *Calocybella* Vizzini, Consiglio & Setti, *Clitolyophyllum* E. Sesli, Vizzini & Contu, *Myochromella* V. Hofst., Clémençon, Moncalvo & Redhead, *Sagaranella* V. Hofst., Clémençon, Moncalvo & Redhead, *Sphagnurus* Redhead & V. Hofst., *Tephrocybella* Picillo, Vizzini & Contu and *Tricholyophyllum* Qing Cai, G. Kost & Zhu L. Yang [2,6,7,8,9,10]; however, a few of them show a poor species diversity worldwide. *Clitolyophyllum* was discovered in 2016, a Turkish species fruiting on the dead bark of *Picea orientalis*. It is mainly characterized by fan-shaped, translucent-striate pileus; decurrent lamellae; lateral, cylindrical to flattened stipe; smooth, inamyloid spores; non-siderophilous basidia and irregular pileipellis [7]. Until now, *Clitolyophyllum* has remained monotypic, and so far, it is known to have only one species in Turkey.

*Calocybe* Kühner ex Donk, typified by *C. gambosa* (Fr.) Donk, was a traditional genus of Lyophyllaceae first discovered by Kühner [11]. Singer’s concept of *Calocybe* included characteristics such as mostly bright-colored pileus, vacuolar pigment, small spores, pileipellis of a cutis or cellular [12]. The taxonomic status of *Calocybe* used to be confused with *Lyophyllum* P. Karst. and *Rugosomyces* Raithelh. [13,14]. Several recent molecular phylogenetic analyses have confirmed *Calocybe* as an independent genus with the merging of *Rugosomyces* [2,3,15,16,17]. At least 57 species can be assigned to *Calocybe* according to Xu et al. [17]; however, the molecular phylogenetic relationships among members of the *Calocybe* were inconsistent with the morphological classification made by Singer [12]. Therefore, the infrageneric classification of *Calocybe* remains in a redelineated position.

The species of Lyophyllaceae are distributed all over the world [12,18,19]. Recently, many new species and new recorded species have also been reported in China [10,15,16,17,20,21,22,23,24,25]. To date, only 14 *Calocybe* species have been reported in China: *Calocybe aurantiaca* X.D. Yu & Jia J. Li, *C. badiofloccosa* J. Z. Xu & Yu Li, *C. carnea* (Bull.) Donk, *C. convexa* X.D. Yu & Jia J. Li, *C. chrysenteron* (Bull.) Singer, *C. decurrens* J. Z. Xu & Yu Li, *C. decolorata* X.D. Yu & Jia J. Li, *C. erminea* J. Z. Xu & Yu Li, *C. gambosa* (Fr.) Donk, *C. gangraenosa* (Fr.) V. Hofst., Moncalvo, Redhead & Vilgalys [=*Lyophyllum leucophaeatum* (P. Karst.) P. Karst.; Index Fungorum], *C. hebelomoides* (Ew. Gerhardt) Læssøe & J.H. Petersen [=*Lyophyllum hebelomoides* (Ew. Gerhardt) Consiglio & Contu; Index Fungorum], *C. ionides* (Bull.) Donk, *C. naucoria* (Murrill) Singer and *C. obscurissima* (A. Pearson) M.M. Moser [15,16,17,24,25,26,27]. However, *Clitolyophyllum* has not been reported in China.

During the survey of macrofungi in northern China, four unique species of the family Lyophyllaceae were newly discovered in the forests dominated by *Acer* sp., *Quercus mongolica* or *Picea crassifolia*. The aim of this study was to describe four new lyophylloid species: *Calocybe fulvipes*, *C. coacta*, *C.* *vinacea* and *Clitolyophyllum umbilicatum* based on morphological features and molecular data. In addition, we explored the species diversity of *Calocybe* based on the previous studies and compiled the information on the *Calocybe* species reported in China.

## 2. Materials and Methods

### 2.1. Specimen Sampling

All the specimens were collected in 2012–2021 from northern China. The collected samples were dried overnight using an electric drying oven at 45 °C and deposited in the Herbarium Mycology of Jilin Agricultural Science and Technology University (HMJU).

### 2.2. Morphological Observation

The macromorphological descriptions were recorded in the field, and images of the basidiocarps were taken in the field with a Canon 80D camera. The Kornerup and Wanscher color description was used to describe color terms, where a specific code is assigned to an observed color [28]. Micromorphology of the specimens was studied with the help of an Olympus BX 53 (Tokyo, Japan) light microscope at 40×, 100×, 400×, 600× and 1000× magnifications (measurements were performed under oil immersion at 1000× magnification). Sections of the dried specimens were mounted in 3% KOH, 1% Congo red and Melzer’s reagent for observations. Cotton blue and iron acetocarmine solutions were used to highlight the siderophilous granulation in the basidia. Factor Q is the value of its length divided by width and Qm is the mean of the Q values. The procedure for scanning electron microscopy (SEM) followed that of Xu et al. [29], and an FEI Quanta 200FE-SEM (JEOL Ltd., Akishima, Japan) was used at an accelerating voltage of 5–10 kV.

### 2.3. DNA Extraction, PCR, Sequencing and Phylogenetic Analyses

Total genomic DNA was extracted using an M5 Fungal Genomic DNA Kit (Mei5 Biotechnology Co., Ltd., Beijing, China) and an Ezup Column Fungi Genomic DNA Purification Kit (Sangon Biotech Co., Ltd., Shanghai, China) according to the manufacturers’ instructions. For the polymerase chain reaction (PCR) amplification, primers ITS1 and ITS4 [30] were used for the ITS region while primer LR0R was paired with LR6 and LR7 in order to obtain sequences for the 28S region [31]. The reactions were performed with the following program: initial denaturation at 94 °C for 5 min (ITS) or 4 min (28S), 33 cycles at 94 °C for 30 s, 46 °C (28S) or 53 °C (ITS) for 30 s or 45 s (28S), and 72 °C for 30 s (ITS) or 40 s (28S), and for terminal elongation, the reaction batches were incubated at 72 °C for 10 min. The PCR products were examined on a 1% agarose gel detected by a JY 600 electrophoresis apparatus (Beijing JUNYI Electrophoresis Co., Ltd., Beijing, China) and then sent to BGI Co., Ltd. (Beijing, China) for sequencing.

### 2.4. Phylogenetic Analyses

The newly generated sequences were compared with the representative ITS and 28S sequences retrieved from GenBank. Species of other genera in Lyophyllaceae were also included according to former phylogenetic studies [2,3,7,10,32], while *Entoloma sericeonitidum* and *Entoloma sericeum* were included as the outgroup. Alignments were generated for the ITS and 28S datasets using MAFFT 7.0 [33] under default conditions and then edited with MEGA 7.0 [34]. The selection of the best-fitting model was completed by ModelFinder [35] based on the Bayesian information criterion (BIC) to provide a substitution model. The GTR + F + I + G4 model was chosen for this purpose. Maximum likelihood (ML) and Bayesian inference (BI) analyses were used to infer the phylogenetic position of the new species. ML estimation was performed through IQ-TREE [36] with 1000 bootstrap replicates [37]. BI phylogeny using Markov chain Monte Carlo (MCMC) methods was carried out with MrBayes 3.2.2 [38]. Two parallel runs were conducted with one cold and four heated chains each for 1,000,000 generations, starting with a random tree. Trees were saved every 1000 generations resulting in broad sampling of 10,001 trees. The initial burn-in was set to 25% (2500 trees). A 50% majority-rule consensus cladogram was computed from the remaining trees to obtain estimates for Bayesian posterior probabilities. The significance threshold was set to >0.95 for Bayesian posterior probability (PP) and >70% for ML bootstrap proportions (BP). All the sequences used in this study are listed in Table 1.

## 3. Results

### 3.1. Phylogenetic Analyses

**Figure 1:**
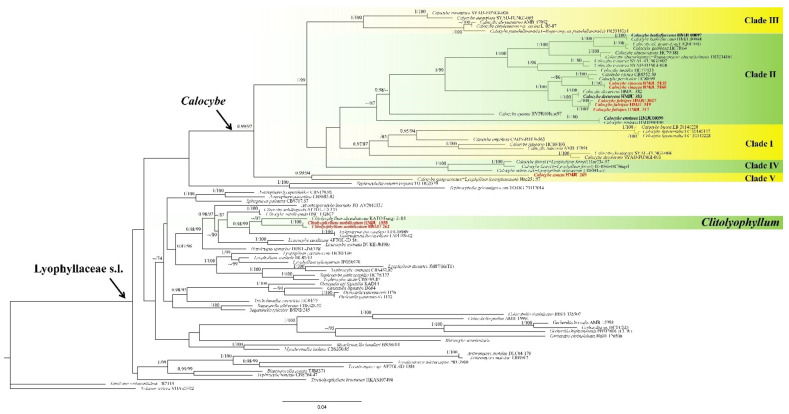
ML and Bayesian phylogenetic analysis of Lyophyllaceae s.l. based on ITS + 28S sequences. The newly generated sequences in black bold and the new species are indicated in red bold.

The combined dataset included 146 sequences, of which 118 were retrieved from GenBank. In the BLAST results, *Calocybe coacta* sequences showed 92.79% similarity to *C. gangraenosa* (=*Lyophyllum leucophaeatum*) (KP192606) with 97% query coverage for ITS and 99.89% similarity to *C. decurrens* (MW444857) with 100% query coverage for 28S; *C. fulvipes* sequences showed 94.96% similarity to *C. carnea* (AF357028) with 79% query coverage for ITS and 99.93% similarity to *C. decurrens* (MW444857) with 100% query coverage for 28S; *C. vinacea* sequences showed 95.73% similarity to *C. carnea* (AF357028) with 86% query coverage for ITS and 99.70% similarity to *C. decurrens* (MW444857) with 100% query coverage for 28S; *Clitolyophyllum umbilicatum* sequences showed 93.17% similarity to *Clitolyophyllum akcaabatense* (MN661004) with 97% query coverage for ITS and 98.68% similarity to *Clitocybe candicans* (AY645055) with 95% query coverage for 28S. Both BI and ML approaches resulted in the same tree topology; as such, only the ML tree is shown, with Bayesian PP values (left) and MLBP values (right) provided near each node (Figure 1). Nodes were annotated if supported by >0.95 Bayesian PP (left) or >70% ML BP (right) values according to Vizzini et al. and Cai et al. [6,10]. In the resulting tree, *Clitolyophyllum umbilicatum* formed a clade with *Clitolyophyllum akcaabatense* Sesli, Vizzini & Contu. The clade was clustered within the core Lyophyllaceae s.l. and occupied an independent phylogenetic position which related to *Tephroderma fuscopallens* Musumeci & Contu. In *Calocybe*, five clades could be recognized corresponding with former phylogenetic studies [15,17]. *Calocybe fulvipes* and *C. vinacea* grouped in clade II related to *C. carnea* (Bull.) Donk, *C. persicolor* (Fr.) Singer and *Calocybe decurrens*, *C. coacta* grouped in clade IV and *C. gangraenosa* (=*Lyophyllum leucophaeatum*) appeared to be the most closely related species.

### 3.2. Taxonomy

**Figure 2:**
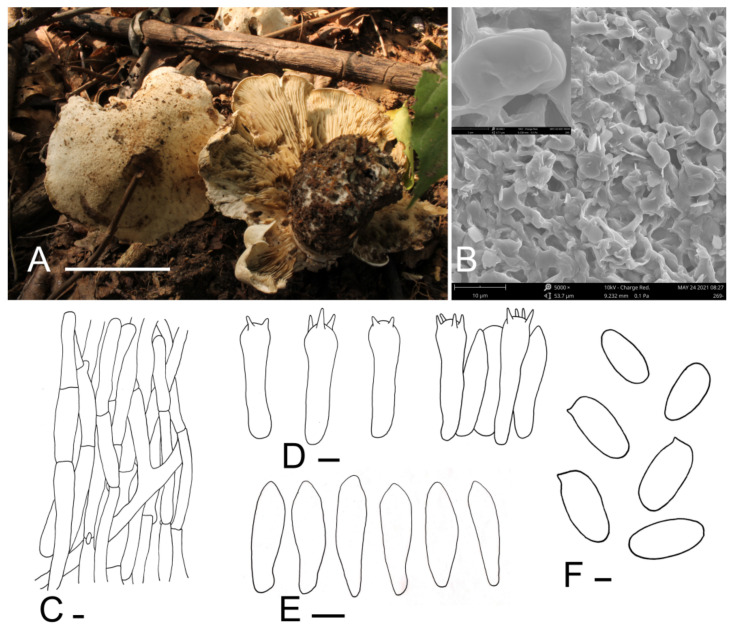
*Calocybe coacta* (HMJU 269, holotype). (**A**) Habitat and basidiocarps; (**B**) SEM images of basidiospores; (**C**) pileipellis; (**D**) basidia; (**E**) hymenial cystidia; (**F**) basidiospores. Scale bar: (**A**) 5 cm; (**C**,**E**) 5 μm; (**F**) 1 μm.

***Calocybe coacta*** J.Z. Xu & Yu Li, sp. nov. (Figure 2).

Mycobank number: MB842022. 

*Diagnosis*: *Calocybe coacta* is distinct from *C.* *gangraenosa* (=*Lyophyllum leucophaeatum*), the most morphologically and molecularly similar species within *Calocybe*, in having a greyish cream felty pileus and smooth spores.

*Holotype*: China, Liaoning Prov., Fuxin City, Haitang Mountain, on soil, under mixed forests dominated by *Acer* sp., 1 September 2012, J.Z. Xu (HMJU 269, holotype).

*Etymology*: “*coacta*”—felted; refers to the felty pileus.

*Description*: Tricholomatoid basidiocarps. *Pileus* 60–115 mm in diam., convex at first, soon becoming planoconvex, with many or few round pits 2–6 mm wide, surface greyish-cream to greyish-yellow (5B3–4B3), felty, not smooth, dry, margin incurved, sometimes irregular. *Lamellae* adnate to decurrent, crowded, 3–5.5 mm wide, concolorous with the pileus or slightly darker towards to the pileus, unchanging when injured, with numerous tiers of lamellulae, edges entire. *Stipe* 40–58 mm long and 20–30 mm wide, broad at the base, concolorous with the pileus, surface longitudinally striate. *Context* white, odor and taste not distinct.

*Basidiospores* (5.7) 6.1–7.8 × (2.7) 2.9–3.9 μm, Q = (1.75) 1.80–2.40 (2.45), Qm = 2.05, oblong, smooth, cyanophilous, inamyloid. *Basidia* (24.1) 25.7–31.7 × 6.3–7.4 (7.6) μm, clavate, tetrasporic, sterigmata up to 4.8 μm long, with numerous internal siderophilous granules. *Hymenial cystidia* 20.0–24.4 × 4.9–6.8 (7.2), fusiform. *Hymenophoral trama* regular, subparallel, hyaline, hyphae cylindrical, thin-walled, 4–18 μm wide, not pigmented. *Pileipellis* a cutis of subparallel, dense, branched cylindrical hyphae, hyphae, 3.3–9.8 μm in diam., thin-walled, not pigmented. *Stipitipellis* composed of parallel, cylindrical, repent, hyaline hyphae, 6.0–17.8 μm wide. *Clamp connections* present.

*Habitat*: Scattered on soil under mixed forests dominated by *Acer* sp.

*Known distribution*: Known only from northeastern China.

*Notes*: *Calocybe coacta* is morphologically similar to species with medium- to large-sized basidiocarps, greyish-cream pileus and non-cellular epicutis in sect. *Calocybe* such as *C*. *pilosella* Floriani & Vizzini, *C*. *gambosa* (Fr.) Donk and *C*. *vasilievae* (Singer) Singer [12]. *Calocybe coacta* differs from *C*. *pilosella* because of its adnate to decurrent lamellae, oblong spores, and *C*. *pilosella* has emarginate lamellae and ovoid to ellipsoid spores. Moreover, *C*. *coacta* has fusiform cystidia, which are absent in *C*. *pilosella* [39]. *C*. *gambosa* and *C*. *vasilievae* are distinguished from *C*. *coacta* by the smooth pileus; the pileus of *C*. *coacta* is felty; also, emarginate lamellae are present in *C*. *gambosa* [26,27,40]. Furthermore, *C*. *coacta* and *C*. *vasilievae* can be differentiated by the size of the stipe (40–58 × 20–30 mm in *C*. *coacta* and 30–50 × 8–10 mm in *C*. *vasilievae*) [41].

**Figure 3:**
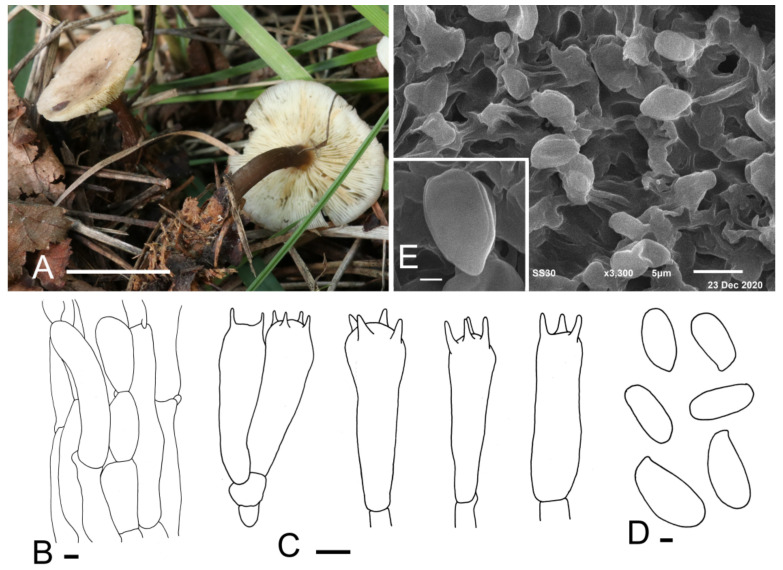
*Calocybe fulvipes* (HMJU 317, holotype). (**A**) Habitat and basidiocarps; (**B**) pileipellis; (**C**) basidia; (**D**) basidiospores; (**E**) SEM images of basidiospores. Scale bar: (**A**) 5 cm; (**B**,**C**) 5 μm; (**D**,**E**) 1 μm.

***Calocybe fulvipes*** J. Z. Xu & Yu Li, sp. nov. (Figure 3).

Mycobank number: MB842023. 

*Diagnosis*: Small to medium basidiocarps, rose-brown to greyish-brown pileus center, adnexed to slightly emarginate lamellae, stone-brown stipe and the absence of cystidia make it unique among the *Calocybe* species.

*Holotype*: China, Jilin Prov., Jilin City, Guoding Moutain, on soil, under mixed forests dominated by *Acer* sp. and *Quercus mongolica*, 9 September 2019, J.Z. Xu (HMJU 317, holotype).

*Etymology*: “*fulvipes*” refers to the stone-brown stipe.

*Description*: *Pileus* 55–70 mm in diam., planoconcave or almost plane with or without a small umbo in the center, center rose-brown to greyish-brown (9D4, 9E3), becoming paler towards the margin, margin brownish-orange (6B3) to brownish-beige (6B2), surface dry, glabrous. *Lamellae* adnexed to slightly emarginate, almost crowded, 1.4–1.8 mm wide, white, greyish-orange (5B3) when bruised, with numerous tiers of lamellulae, edges entire. *Stipe* 50–75 mm long and 6–11 mm wide, cylindrical, slightly broad at the base, solid; surface stone-brown (7F5) to deep mahogany (7F8), fibrillose. *Context* white, odor and taste not distinct.

*Basidiospores* (4.5) 5.0–6.4 (7.5) × (2.1) 2.4–3.2 (4.3) μm, Q = (1.67) 1.78–2.42 (2.75), Qm = 2.12, oblong, sometimes bacilliform, smooth, cyanophilous, inamyloid. *Basidia* 14.7–20.2 (20.6) × (3.4) 4.7–6.1 (6.5) μm, clavate, sometimes cylindrical, tetrasporic, sterigmata up to 3 μm long, with numerous internal siderophilous granules. *Cystidia* absent. *Hymenophoral trama* regular, parallel, hyaline, hyphae cylindrical, 3–13 μm wide, thin-walled, not pigmented. *Pileipellis* a cutis of subparallel, dense, repent, cylindrical hyphae, 3.5–12.9 μm in diam., thin-walled, not pigmented. *Stipitipellis* composed of subparallel, cylindrical, repent, hyaline hyphae, 5.0–14.2 μm wide. *Clamp connections* present.

*Habitat*: Scattered on soil under mixed forests dominated by *Acer* sp. and *Quercus mongolica*.

*Known distribution*: So far, only known from northeastern China.

*Additional material examined*: China, Jilin Prov., Yanbian Korean Autonomous Prefecture, Longjing City, 16 September 2019, J.Z. Xu (HMJU 319); Jilin City, Guoding Mountain, 5 September 2013, J.Z. Xu (HMJU 2912, HMJU 3027).

*Note*: The key characteristics of *C*. *fulvipes* are its rose-brown to greyish-brown pileus, stone-brown stipe and non-cellular epicutis, which suggest that it should be in sect. Carneoviolaceae [12]. In that section, *C*. *carnea* resembles *C*. *fulvipes* due to the light carneous pileus and similar spore size. They can be differentiated by the color of stipe and the shape of the pileus. *Calocybe carnea* produces a convex pileus and a white stipe, while *C*. *fulvipes* has a plane pileus and a brown stipe [41].

**Figure 4:**
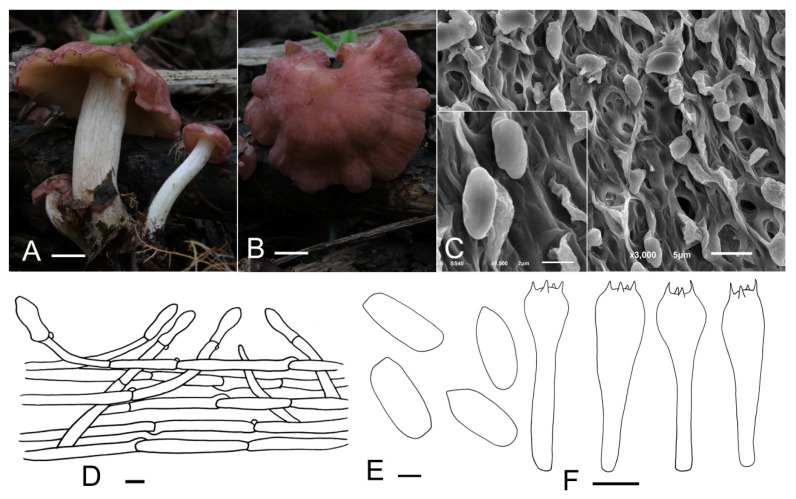
*Calocybe vinacea* (HMJU 5135, holotype). (**A**,**B**) Habitat and basidiocarps; (**C**) SEM images of basidiospores; (**D**) pileipellis; (**E**) basidiospores; (**F**) basidia. Scale bar: (**A**,**B**) 1 cm; (**D**) 10 μm; (**E**) 1 μm; (**F**) 5 μm.

***Calocybe vinacea*** J. Z. Xu & Yu Li, sp. nov. (Figure 4).

Mycobank number: MB842024. 

*Diagnosis*: Characterized by its slender tricholomatoid basidiocarps of small to medium size, pastel red to dull-red pileus with densely white appendages, adnate to subdecurrent lamellae, stipe with densely mycelium-like appendages, oblong spores and clavate or subcapitate terminal elements in the pileipellis.

*Holotype*: China, Jilin Prov., Baishan City, Manjiang Town, on the humus in broad-leaved forests, 1 September 2021, J.Z. Xu (HMJU 5135, holotype).

*Etymology*: “*vinacea*”—wine-colored, refers to the dull-red pileus.

*Description*: Basidiocarps slenderly tricholomatoid. *Pileus* 15–55 mm in diam., convex or hemispherical at first, then planoconvex, with a broad obtuse umbo, surface pastel red to dull-red (10A5–10C3), slightly hygrophanous, not striate, with densely white pruina becoming sparser at maturity; margin incurved, even, entire when young, sometimes irregular, wavy, undulating, strongly and deeply plurilobed to subpetaloid at mature stages. *Lamellae* adnate to subdecurrent, crowded, 0.5–3 mm wide, yellowish-white (4A2), with numerous tiers of lamellulae; edges entire or slightly eroded. *Stipe* 26–57 mm long and 6–17 mm wide, central, cylindrical, subequal, surface white (10A1), longitudinally striate, with appendages the same as the pileus, subtended by abundant and thin whitish rhizoids at the base. *Context* thin, fleshy.

*Basidiospores* (3.5) 4.2–5.3 (5.6) × 2.2–2.8 μm, Q = (1.39) 1.55–2.24 (2.35), Qm = 1.93, oblong, smooth, cyanophilous, inamyloid. *Basidia* (15.0) 16.3–23.0 (23.8) × (3.6) 4.0–5.8 μm, clavate, tetrasporic, with numerous internal siderophilous granules. *Hymenial cystidia* not observed. *Hymenophoral trama* regular composed of parallel, cylindrical, hyaline, thin-walled, 2–11 μm wide hyphae. *Pileipellis* a cutis of subparallel, cylindrical, hyaline hyphae, hyphae 2–14 μm wide, thin-walled; terminal elements, (18.4) 20.6–31.5 (38.9) × (5.0) 5.4–8.2 (8.9) μm, clavate, subcapitate, thin-walled, hyaline. *Stipitipellis* regular consisting of parallel, cylindrical, hyaline hyphae, hyphae 2–11 μm wide, thin-walled. *Clamp connections* present.

*Habitat*: Scattered on the humus in broad-leaved forests.

*Known distribution*: Known only from northeastern China.

*Additional material examined*: China, Jilin Prov., Baishan City, Manjiang Town, on the humus in broad-leaved forests, 1 September 2021, J.Z. Xu (HMJU 5160).

*Notes*: The pastel red to dull-red pileus, smooth spores and pileipellis of the cutis type suggests that *C*. *vinacea* should be in sect. Carneoviolaceae [12]. The unique pileus color, deeply plurilobed to subpetaloid margin in age, longitudinally striate stipe with densely white pruina overall and clavate or subcapitate terminal elements in the pileipellis apparently separate it from other species within the section. *C*. *rubra* Rick ex Redhead & Singer and *C*. *bipigmentata* Singer which used to be placed in sect. Pseudoflammulae by Singer resemble *C*. *vinacea* in the pileus color; however, *C*. *rubra* differs in a carnea-red and narrow stipe of only 3 mm wide [42,43]. *C*. *bipigmentata* is mainly different from *C*. *vinacea* in having ochraceous lamellae, a glabrous, ochraceous stipe and narrow-fusoid cheilocystidia, which lacking in *C*. *vinacea* [44].

**Figure 5:**
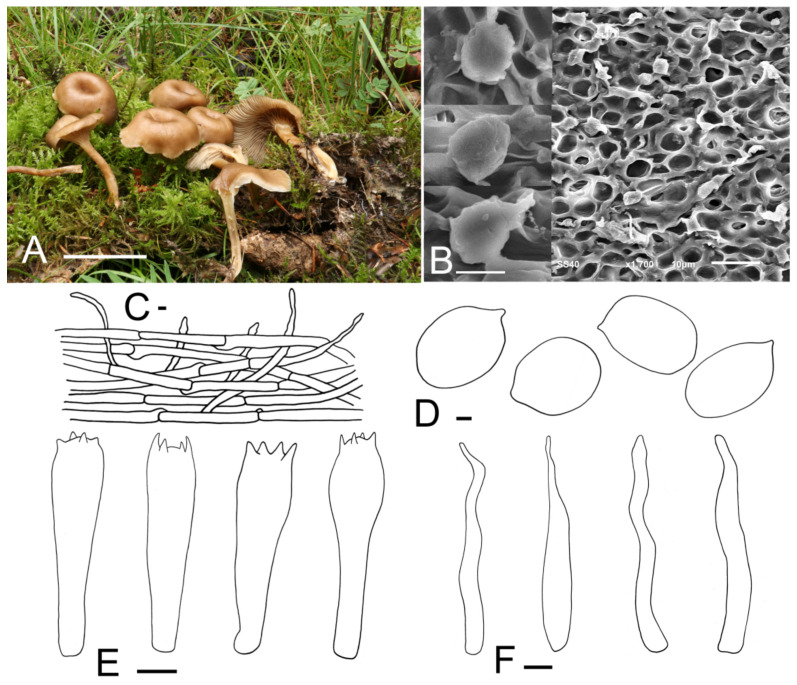
*Clitolyophyllum umbilicata* (HMJU 262, holotype). (**A**) Habitat and basidiocarps; (**B**) SEM images of basidiospores; (**C**) pileipellis; (**D**) basidiospores; (**E**) basidia; (**F**) terminal elements in the pileipellis. Scale bar: (**A**) 5 cm; (**C**) 10 μm; (**D**) 1 μm; (**B**,**E**,**F**) 5 μm.

***Clitolyophyllum umbilicatum*** J. Z. Xu & Yu Li, sp. nov. (Figure 5).

Mycobank number: MB842025. 

*Diagnosis*: It is distinguished by the omphalioid or clitocyboid habit, umbilicate pileus, central stipe, smooth, inamyloid spores and subregular pileipellis.

*Holotype*: China, Gansu Prov., Zhangye City, Yugur Autonomous County of Sunan, Xishui Forest Farm, in the moss, under a forest dominated by *Picea crassifolia*, 10 August 2018, J.Z. Xu (HMJU 262, holotype).

*Etymology*: “*umbilicatum*” refers to the umbilicate pileus.

*Description*: Basidiocarps omphalioid or clitocyboid. *Pileus* 38–62 mm in diam., deeply depressed, dark orange to light brown (6B6–6D7), surface smooth, slightly hygrophanous; margin incurved to straight, white to greyish-orange (6A1–6B3), radially striate, slightly wavy with age. *Lamellae* decurrent, moderately crowded, 4–8 mm wide, thin, color slightly paler than the pileus (6B2–6B4), with numerous tiers of lamellulae, edges entire, even. *Stipe* 52–74 mm long and 5–9 mm wide, central, cylindrical or slightly compressed, equal or slightly tapering towards the apex, hollow, surface orange-grey to light brown (6B2–6D7), longitudinally striate, often with white appendages at the apex. *Context* thin, fleshy, slightly paler than the pileus.

*Basidiospores* (4.9) 5.3–8.0 (8.8) × (3.8) 4.0–5.7 μm, Q = (1.14) 1.20–1.54 (1.62), Qm = 1.36, subglobose-ellipsoid, smooth, inamyloid, cyanophilic. *Basidia* (20.0) 21.2–28.7 (29.0) × (5.1) 5.7–8.0 (8.3) μm, narrowly clavate or clavate, 2–4 spores, siderophilous granulations not obviously observed. *Hymenophoral trama* regular, consisting of subcylindrical, subinflated, thin-walled, 2.2–18.1 μm wide hyphae. *Hymenial cystidia* not observed. *Pileipellis* a cutis composed of subparallel, dense, cylindrical hyphae, hyphae 2.1–18.9 μm wide, thin-walled; terminal elements, (30.2) 33.8–50.6 (54.5) × (3.0) 3.6–5.7 (7.4) μm, irregular, cylindrical, flexuose, thin-walled, pileocystidia-like. *Stipitipellis* made up of regularly parallel, cylindrical, hyaline hyphae, hyphae 2.3–16.4 μm wide. *Clamp connections* present.

*Habitat*: Scattered in the moss under a forest dominated by *Picea crassifolia.*

*Known distribution*: Known only from northwestern China.

*Additional material examined*: China, Gansu Prov., Zhangye City, Yugur Autonomous County of Sunan, Xishui Forest Farm, in the moss under a forest dominated by *Picea crassifolia*, 10 August 2018, J.Z. Xu (HMJU 1558).

*Note*: *Clitolyophyllum akcaabatense* shares a few characteristics with *C. umbilicatum* such as the similarly sized pileus, decurrent lamellae, hollow stipe, similar smooth, inamyloid basidiospores, non-siderophilous basidia, absence of hymenial cystidia and the pileocystidia-like terminal elements in the pileipellis. However, *C. akcaabatense* is distinguished from *C*. *umbilicatum* in having a fan-shaped pileus with a small depression where it is connected to the stipe, a lateral stipe that tapers towards the base, with lower two thirds covered with a typical white to creamy, woolly mycelium [7]. Microscopically, *C*. *akcaabatense* is mainly different from *C*. *umbilicatum* in having ellipsoid-fusoid, sublacrymoid spores with Q = 1.58–1.78 [spores subglobose-ellipsoid, Q = (1.14) 1.20–1.54 (1.62) in *C. umbilicatum*] and irregular pileipellis [7].

## 4. Discussion

In the combined ITS and LSU phylogenetic analysis (Figure 1), Lyophyllaceae s.l. appear to be polyphyletic, which is consistent with previous studies [2,3,5,10]. The new species described in this study occupy independent positions. *Clitolyophyllum umbilicatum* formed a distinct clade with *Clitolyophyllum akcaabatense*, but morphologically, *C. akcaabatense* can be distinguished from *C. umbilicatum* by a fan-shaped pileus with a small depression, a lateral stipe that tapers to the base and ellipsoid-fusoid, sublacrymoid spores. Furthermore, *Clitolyophyllum umbilicatum* grows on the moss under forests dominated by *Picea crassifolia*, while *C. akcaabatense* grows on the dead bark of *Picea orientalis* together with mosses.

Phylogenetically, *Calocybe fulvipes* clusters in a single clade sister to a clade of *C. decurrens*; however, they can be easily distinguished from one another by morphology. *Calocybe fulvipes* has a glabrous pileus, adnexed to slightly emarginate lamellae and a stone-brown stipe which does not change with age while *C. decurrens* has a carneous, slightly pruinose pileus, decurrent lamellae and a pastel stipe that turns brownish-red or greyish-brown when mature [17]. In addition, *C. fulvipes* grows on soil under mixed forests dominated by *Acer* sp. and *Quercus mongolica*, while *C. decurrens* grows on soil under mixed forests dominated by *Salix* sp. *Calocybe vinacea* is a group in clade II and is related to *C. carnea* and *C. persicolor*. The combination of the pastel red to dull-red pileus with densely spaced white pruina and white stipe with the same surface are unique to *C. vinacea,* and are unknown in other *Calocybe* species. *Calocybe coacta* is related to *C. gangraenosa (Lyophyllum leucophaeatum*), while *C. coacta* is distinct from *C. gangraenosa* in having a greyish-cream felty pileus and smooth spores, while *C. gangraenosa* produces a whitish to brownish-grey (when matured) pileus and spores ornamented with warts [27,43].

*Calocybebuxea* (Maire) Raithelh. and *C. hypoxantha* (Joss. & Riousset) Bon. were placed in clade I in single-gene analysis based on ITS and 28S by Li et al. but were not present in the combined analysis [15]. In the present study, they were located in clade II. Hence, the location of the two species may require more material, mainly, both taxa sampled and other gene fragments, to determine.

In our research on the species of *Calocybe* in China, we failed to get the type specimens and corresponding morphological description of *C. hebelomoides*, and could not confirm the existence of this species in China. The taxonomic treatments of *C. hebelomoides* from China should be performed on the basis of additional detailed morphological investigations in later studies. A key for the other 16 *Calocybe* species reported from China is provided below:
1. Basidiocarps medium to large, pileus usually more than 6 cm diam, stipe more than 3.5 cm long21’. Basidiocarps small, pileus usually less than 6 cm diam, stipe less than 3.5 cm long62. Lamellae sinuate32’. Lamellae not sinuate43. Pileus bruised blue, spore non-smooth*C. gangraenosa*3’. Pileus bruised unchanged, spore smooth*C. gambosa*4. Lamellae adnexed to slightly emarginate, lamellae less than 3 mm width*C. fulvipes*4’. Lamellae decurrent, lamellae more than 3 mm width55. Stipe base broad, lamellae greyish orange when bruised, cystidia present*C. coacta*5’. Stipe base narrow, lamellae unchanged when bruised, cystidia absent*C. decurrens*6. Lamellae bruised blue*C. decolorata*6’. Lamellae unchanged when bruised77. Stipe base with white pubescence*C. badiofloccosa*7’. Stipe base without white pubescence88. Lamellae decurrent, pileus left plane and slightly depressed98’. Lamellae not decurrent, pileus left convex109. Pileus margin regular, stipe apex with whitish pruina*C. erminea*9’. Pileus margin flexuous, stipe apex without whitish pruina*C. aurantiaca*10. Lamellae sinuate, pileus with purple tones.*C. ionides*10’. Lamellae adnate, pileus without purple tones1111. Lamellae white1211’. Lamellae not white 1312. Pileus yellow, pileus surface fibrillose, basidiospores less than 5 μm length*C. convexa*12’. Pileus pink, pileus surface smooth, basidiospores more than 5 μm length*C. carnea*13. Lamellae with yellow tones1413’. Lamellae with taupe tones*C. obscurissima*14. Pileus diam more than 5.5 cm, Stipe with pruinose at apex*C. chrysenteron*14’. Pileus diam less than 5.5 cm, Stipe without pruinose at apex1515. Pileus surface pastel red to dullred, basidiospores more than 4 μm length*C. vinacea*15’. Pileus surface reddish yellow, basidiospores less than 4 μm length*C. naucoria*

## Figures and Tables

**Table 1:** Taxa names, accession numbers and the corresponding GenBank accession numbers of the taxa used in the molecular analyses. The newly generated sequences are in black bold.

Species	Collection	GenBank Accession Numbers	
		ITS	28S
*Arthromyces matolae*	TJB9912	EU708338	EU708335
*Arthromyces matolae*	DLC04-170	EU708339	EU708336
*Asterophora lycoperdoides*	CBS170.86	AF357037	AF223190
*Asterophora parasitica*	CBS683.82	AF357038	AF223191
*Atractosporocybe inornata*	TO AV201012d	KJ680993	KJ681046
*Blastosporella zonata*	TJB8371	EU708340	EU708337
*Calocybe aurantiaca*	SYAU-FUNGI-005	KU528828	KU528833
*Calocybe aurantiaca*	SYAU:FUNGI 006	NR156304	NG058937
***Calocybe badiofloccosa***	**HMJU00099**	**OK649912**	**OK649882**
*Calocybe badiofloccosa*	HMJU00100	MN172331	MN172333
*Calocybe buxea*	EB 20140228	KP885633	KP885625
*Calocybe carnea*	CBS552.50	AF357028	AF223178
*Calocybe cf. graveolens*	FR2014043	KP192609	–
*Calocybe chrysenteron*	AMB 17092	KP885639	KP885628
*Calocybe chrysenteron var.* *cerina*	L 05-87	KP885640	KP885629
***Calocybe coata***	**HMJU269**	**OK649907**	**OL687156**
*Calocybe convexa*	SYAU-FUNGI-007	KU528826	KU528830
*Calocybe convexa*	SYAU:FUNGI 008	NR156303	NG058936
*Calocybe cyanea*	RVPR10June97	–	AF261400
*Calocybe decolorata*	SYAU-FUNGI-003	KU528824	KU528834
*Calocybe decolorata*	SYAU:FUNGI 004	NR_156302	NG_058938
*Calocybe decurrens*	HMJU 382	MT080028	MW444857
***Calocybe decurrens***	**HMJU 383**	**OK649913**	**OK649883**
***Calocybe erminea***	**HMJU00097**	**OK649911**	**OK649881**
*Calocybe erminea*	HMJU00098	MN172332	MN172334
***Calocybe fulvipes***	**HMJU 317**	**MT071590**	**OK649878**
***Calocybe fulvipes***	**HMJU 319**	**MW406907**	**OK649879**
***Calocybe fulvipes***	**HMJU 3027**	**OK649910**	**OK649880**
*Calocybe gambosa*	HC78/64	AF357027	AF223177
*Calocybe gangraenosa*	Hae251.97	AF357032	AF223202
*Calocybe hypoxantha*	EC 20140117	KP885634	KP885626
*Calocybe hypoxantha*	EC 20140228	KP885635	–
*Calocybe ionides*	HC77/133	AF357029	AF223179
*Calocybe naucoria*	AMB 17094	KP885642	KP885630
*Calocybe naucoria*	HC80/103	AF357030	AF223180
*Calocybe obscurissima*	HC79/181	AF357031	AF223181
*Calocybe onychina*	CAON-RH19-563	MW084664	MW084704
*Calocybe persicolor*	HC80/99	AF357026	AF223176
***Calocybe vinacea***	**HMJU5135**	**OK649908**	**OK649876**
***Calocybe vinacea***	**HMJU5160**	**OK649909**	**OK649877**
*Calocybella dominicana*	JBSD 126507	KY363575	KY363577
*Calocybella pudica*	AMB 15994	KP858000	KP858005
*Clitocybe subditopoda*	AFTOL-ID 533	DQ202269	AY691889
*Clitocybe subditopoda*	OSC 112607	EU697244	EU852807
*Clitolyophyllum akcaabatense*	KATO Fungi 3184	KT934393	KT934394
***Clitolyophyllum umbilicatum***	**HMJU 262**	**OK649905**	**OK649873**
***Clitolyophyllum umbilicatum***	**HMJU 1558**	**OK649906**	**OK649874**
*Entoloma sericeonitidum*	TB7144	EF421108	AF261315
*Gerhardtia borealis*	AMB 15993	KP858004	KP858009
*Gerhardtia citrinolobata*	JBSD 126508	KY363576	KY363578
*Gerhardtia highlandensis*	PBM2806 (CUW)	GU734744	EF535275
*Gerhardtia sp.*	HC01/025	EF421103	EF421091
*Hypsizygus ulmarius*	DUKE-JM/HW	EF421105	AF042584
*Leucocybe candicans*	AFTOL-ID 541	DQ202268	AY645055
*Leucocybe connata*	DUKE-JM90c	EF421104	AF042590
*Lyophyllum caerulescens*	HC80/140	AF357052	AF223209
*Lyophyllum decastes*	JM87/16(T1)	AF357059	AF042583
*Lyophyllum favrei*	IE-BSG-HC96cp4	EF421102	AF223184
*Lyophyllum favrei*	Hae234.97	AF357034	AF223183
*Lyophyllum leucophaeatum*	Hae251.97	AF357032	AF223202
*Lyophyllum ochraceum*	BSI94.cp1	AF357033	AF223185
*Lyophyllum semitale*	HC85/13	AF357049	AF042581
*Lyophyllum sykosporum*	IFO30978	AF357050	AF223208
*Myochromella boudieri*	BSI96/84	AF357047	DQ825430
*Myochromella inolens*	CBS330.85	AF357045	AF223201
*Nolanea sericea*	VHAs03/02	DQ367430	DQ367423
*Ossicaulis aff. lignatilis*	KAD14	MG663236	MT237454
*Ossicaulis lignatilis*	D604	DQ825426	AF261397
*Ossicaulis yunnanensis*	IJ152	KY411962	KY411960
*Ossicaulis yunnanensis*	IH26	KY411961	KY411959
*Rugosomyces obscurissimus*	FR2014101	KP192650	–
*Rugosomyces pseudoflammulus*	FR2014054	KP192579	–
*Rhizocybe vermicularis*	AH33078	KJ681032	–
*Sagaranella gibberosa*	CBS328.50	AF357041	AF223197
*Sagaranella tylicolor*	BSI92/245	AF357040	AF223195
*Sphagnurus palustris*	CBS717.87	AF357044	AF223200
*Tephrocybe ambusta*	CBS452.87	AF357057	AF223216
*Tephrocybe anthracophila*	HC79/132	AF357055	AF223212
*Tephrocybe atrata*	CBS709.87	AF357053	AF223210
*Tephrocybe rancida*	CBS204.47	AF357025	AF223203
*Tephrocybella constrictospora*	TO HG3329	MF614962	MF614963
*Tephrocybella griseonigrescens*	TO:HG 21112014	NR137975	KR476785
*Tephroderma fuscopallens*	EM4789-12	KJ701326	KJ701332
*Tephroderma fuscopallens*	LUG18989	KJ701327	KJ701333
*Termitomyces microcarpus*	PRU3900	AF357023	AF042587
*Termitomyces sp.*	AFTOL-ID 1384	DQ494698	DQ110875
*Tricholomella constricta*	HC84/75	AF357036	AF223188
*Tricholyophyllum brunneum*	HKAS107494	MT705717	MT734655
